# Driver Steering Intention Prediction for Human-Machine Shared Systems of Intelligent Vehicles Based on CNN-GRU Network

**DOI:** 10.3390/s25103224

**Published:** 2025-05-20

**Authors:** Chen Zhou, Fan Zhang, Edric John Cruz Nacpil, Zheng Wang, Fei-Xiang Xu

**Affiliations:** 1 Information and Control Engineering, China University of Mining and Technology, Xuzhou 221000, China; zc111@cumt.edu.cn (C.Z.); 14214515@cumt.edu.cn (F.Z.); 2 Fujian Key Laboratory of Green Intelligent Drive and Transmission for Mobile Machinery, Huaqiao University, Xiamen 361021, China; 3 Advanced Institute of Nano Technology, Sungkyunkwan University, Suwon 16228, Republic of Korea; enacpil@uci.edu; 4 Institute of Industrial Science, The University of Tokyo, Tokyo 113-8654, Japan; z-wang@iis.u-tokyo.ac.jp

**Keywords:** shared steering control, driver intention prediction, CNN-GRU network

## Abstract

In order to mitigate human-machine conflicts and optimize shared control strategy in advance, it is essential for the shared control system to understand and predict driver behavior. This paper proposes a method for predicting driver steering intention with a CNN-GRU hybrid machine learning model. The convolutional neural network (CNN) layer extracts features from the stochastic driver behavior, which is input to the gated-recurrent-unit (GRU) layer. And the driver’s steering intention is forecasted based on the GRU model. Our study was conducted using a driving simulator to observe the lateral control behaviors of 18 participants in four different driving circumstances. Finally, the efficiency of the suggested prediction approach was evaluated employing long-short-term-memory, GRU, CNN, Transformer, and back propagation networks. Experimental results demonstrated that the proposed CNN-GRU model performs significantly better than baseline models. Compared with the GRU network, the CNN-GRU network reduced the RMSE, MAE, and MAPE of the driver’s input torque by 33.22%, 32.33%, and 35.86%, respectively. The proposed prediction method also possesses adaptability to different driver behaviors.

## 1. Introduction

New types of automated driving techniques bring enormous opportunities and challenges to the automotive industry [1,2]. According to various factors such as legal and ethical considerations, a fully autonomous vehicle is difficult to achieve in the short term. In addition to the challenge of determining driver preferences regarding vehicle operation, it is currently not feasible for drivers to detach themselves completely from the driving process [3]. Therefore, human-machine shared control is necessary to transition from semi-automation to full automation [4,5].

Human-machine shared steering control methods can be classified into two types: haptic shared control and indirect shared steering [6,7]. Compared with the indirect shared steering system, the haptic shared steering system enables the driver with haptic torque feedback from the automation system, via the steering wheel [8,9]. This method reduces the burden on the driver by continuously adjusting the torque on the driver [10]. Drivers can also exert greater force on the steering wheel to overturn the decisions made by the automation system whenever they choose [11]. Therefore, the haptic shared steering system has been extensively applied in vehicles [12].

Haptic shared steering system essentially predicts the driver’s steering intention [13]. The haptic steering system uses the predictions to optimize shared control strategies and mitigate human-machine conflicts [14]. Predicting driver intention also smoothens the transfer of control from the vehicle to the driver [15]. For example, in emergency situations, the haptic steering system can determine the capability of the driver to take over by predicting driver intention [16].

There are many driver intention prediction tasks in different environments. This study addresses the driver’s intention prediction during the steering process, in which the driver’s behavior is continuously changing. This behavior is directly influenced by the driver’s steering intention. Since the steering torque applied by the driver varies depending on the changes in behavior, the haptic shared steering system can estimate the intention by predicting the steering torque. There are two main categories of methods used to predict driver intention: model-based methods and machine learning methods. Model-based methods have notable interpretability and a high level of security [17]. Researchers have combined a linear quadratic regulator and a Kalman filter to predict the steering torque based on a pre-assumed driver model in [18]. Others have utilized an unscented Kalman filter to predict steering torque with a constant turning rate and acceleration mode in [19]. To ensure the prediction accuracy, these methods require previous information and detailed mathematical models of the system [20]. However, obtaining precise physical parameters is often difficult due to the stochastic human driving behavior, especially in highly dynamic driving scenarios. Dong et al. [21] validated that the stochastic variations of driver behaviors can significantly affect the accuracy of driver behavior modeling. Therefore, it is urgent to develop an efficient approach that can predict human driver behavior accurately.

In recent years, machine learning methods have been drawing broad attention due to their freeform model, such as Hidden Markov Model, Bayesian network, artificial neural network, and reinforcement learning [22]. The Hidden Markov Model is capable of predicting drivers’ behaviors through probabilistic inference. However, it requires predefined parameters of different driver states, which limits its generalization ability [23]. A filter based on dynamic Bayesian networks is proposed to predict driver behavior, but this method has a high computational complexity and still relies on pre-set assumptions [24]. Moreover, the artificial neural network can automatically extract features from the original input data with an end-to-end modeling approach. However, it is difficult for artificial neural networks to handle multidimensional inputs owing to their simple structures [25]. Reinforcement learning aims to maximize long-term rewards through interactions with the environment, which is more suitable for optimizing long-term decisions. Nonetheless, it may struggle to react quickly and adapt to complex driving scenarios when predicting the driver’s short-term intentions. Additionally, the personalized nature of driver behaviors makes the design of the reinforcement learning reward function more challenging.

Although past modeling methods are capable of predicting driver intentions to some extent, it is challenging to predict multi-step intentions in nonlinear human-machine systems [26]. In the shared steering systems of intelligent vehicles, high nonlinearity stems from the pronounced haptic interaction between the driver and the automation system [27,28]. Nevertheless, the long-short-term-memory (LSTM) network can predict longer steps in nonlinear situations because LSTM networks are specifically designed to analyze temporal data. Therefore, the LSTM network suitably encodes the behavioral characteristics of the driver to predict in the time domain [29,30]. Although the LSTM network can finish the prediction of time series data, it is difficult for the system to achieve fast prediction speed with a complex architecture. This is achieved with gate mechanisms that increase memory storage through the elimination of dedicated memory units [31]. Unlike the LSTM network, the GRU network is capable of efficiently predicting driver intention with a simplified architecture [32].

In human-machine shared control systems, the driver’s output torque exhibits strong nonlinearity due to the intervention of haptic guidance torque. Moreover, the input includes the haptic guidance torque and multi-source information, such as vehicle states and driver near and far points, resulting in a multi-dimensional feature set. Due to its strong ability to resist interference and process multidimensional information during feature extraction, a convolutional neural network (CNN) performs well in handling complex input data. As a result, a front-end feature extractor is incorporated with a CNN in this study. Researchers have demonstrated the CNN as an effective feature extractor in various fields [33,34]. On one hand, the convolutional layers of the CNN model are sparsely connected to the input with individual filters to extract the features effectively [35]. On the other hand, a pooling layer assists the CNN with retaining all features through reduced parameters [36].

Inspired by previous work, a CNN-GRU network was designed in this study to anticipate drivers’ steering intentions. The CNN model extracted features from the driver behavior data, whereas the GRU model predicted the driver’s steering intention. Experiments were conducted using a driving simulator, involving 18 participants, to analyze the lateral control behaviors exhibited by the drivers. Four experimental conditions were conducted to account for different driver steering tendencies. The experimental results verified the efficacy of the CNN-GRU network in different haptic torque scenarios during driver interactions.

The main contributions of the proposed methods can be summarized as follows:

1. The highly nonlinear and multidimensional input features (the haptic guidance torque, vehicle state, and driver near and far points) of the driver steering intention prediction model are extracted based on the CNN module.
2. The CNN and GRU networks are combined to predict the steering intention of drivers accurately by considering the haptic interaction between the driver and the automation system. Moreover, the proposed driver intention prediction strategy is validated through driving simulator experiments.

This publication provides further information that supports this conclusion: Section 2 provides a detailed description of the experimental platform, equipment, and situations. Section 3 explains the method for predicting driver steering intentions using the CNN-GRU model. In Section 4, we discuss the experimental results. In Section 5, the conclusions are finally drawn.

## 2. Experimental Design

The current study carried out driving simulator experiments to capture the driver behavior data. The proposed method for predicting steering intentions was formulated using the gathered data. What follows are descriptions of the test subjects, equipment, and experimental conditions involved in the data collection.

### 2.1. Participants

To obtain the driver steering behaviors, 18 participants consisting of 16 males and 2 females (mean age = 22 years, standard deviation (SD) = 1.8 years) were recruited for the experiments. Each participant owned a valid Japanese driver’s license with driving experience (mean = 2.7 years, SD =2.6 years). These participants drove about 6000 km each year on average. In addition, these participants usually drove on urban roads and expressways. While the gender ratio in the sample is uneven, it is reasonable because there are few significant gender differences in lane-changing behaviors, which are mainly influenced by drivers’ personal habits and response abilities. The University of Tokyo’s Ethics and Safety Office for Life Sciences sanctioned the experiment (No. 12 in 2017).

### 2.2. Apparatus

**Figure 1:**
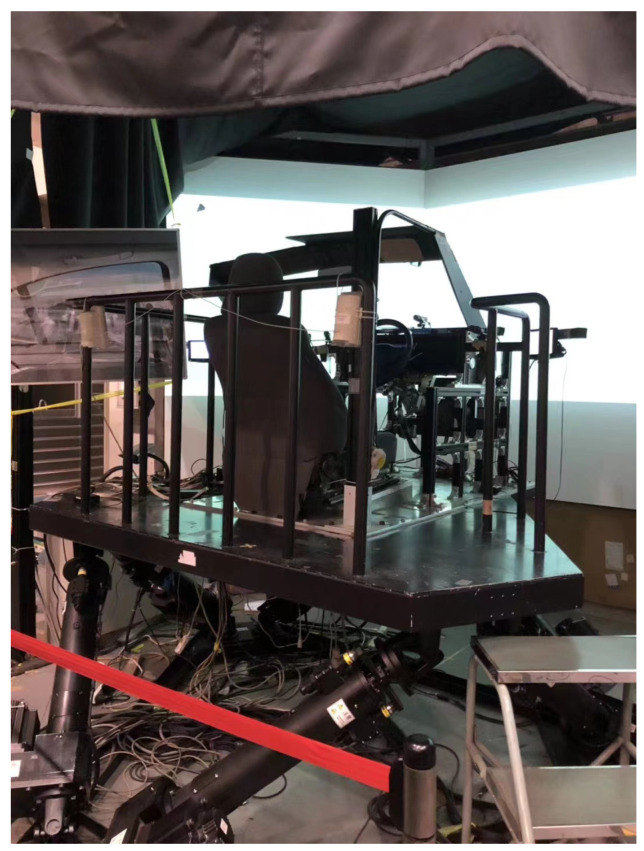
Driving simulator [32].

To guarantee the precision of the experimental results, the experiment utilized a driving simulator [32] to simulate a real-life road setting, as shown in Figure 1. This driving simulator can be divided into two parts: the D3sim software platform (Ver.6) and actual hardware modules. The D3sim software platform on the host computer was used to simulate the driving scenarios and vehicle movement, and it was developed by Mitsubishi Precision Co., Ltd. (Tokyo, Japan) The actual hardware modules consisted of a steering wheel, brake pedal, accelerator pedal, instrument panel, stereo speakers, 140 degrees viewer, and 6 degrees motion platform. The 140-degree viewer can be used to provide drivers with a broad driving view. The 6 degrees motion platform was utilized to replicate driving conditions by generating high-frequency vibrations [37]. This motion platform enabled drivers to have a better feeling of the road and vehicle status. In addition, the sound of the engine was simulated for drivers through two stereo speakers. These hardware modules and D3sim software with realistic driving scenarios make drivers approach real behaviors, which strengthens the driving simulator. In the driving simulator experiments, the driver’s input command with the real steering wheel, brake pedal, and accelerator pedal is transferred to the host computer [38]. Then the vehicle is manipulated from the driver’s command on the D3sim software. Meanwhile, the human-machine shared controller calculates and offers precise haptic torque input to drivers. Finally, the human-machine shared steering system is realized in the driving simulator.

### 2.3. Experimental Conditions and Scenario

Table 1 lists four experimental conditions accounting for different driver states and haptic guidance configurations. The experiment was conducted under attentive and distracted states with two forms of haptic guidance: fixed gain (HG-Fixed) and adaptive gain (HG-Adaptive).

An adaptive gain haptic guidance system was designed to adjust the allocation of human-machine authority. The torque for haptic guidance is calculated using the following formula:

(1) $$T_{h}=K(a_{1}e_{y(near)}+a_{2}e_{\theta (far)})$$

where $e_{y(near)}$, $e_{\theta (far)}$ represents the visual information from the driver; $a_{1}$ and $a_{2}$ are the constant gains for the $e_{y(near)}$ and $e_{\theta (far)}$, and their values are respectively set to 0.19 and 3.8. K is a constant factor that determines the magnitude of the haptic guiding torque.

This system uses a Myo armband to collect surface electromyography signals from the driver’s forearm. To prepare the surface electromyography signals for further processing, it calculates the root mean square of the activation signals from the stainless-steel armband sensors. The driver’s grip force is normalized against each participant’s maximum surface electromyographic signals. Then, based on the normalized surface Electromyography signals, it adjusts the proportion between the total steering torque and the tactile guidance torque. When the grip force increases, the system reduces the proportion of the haptic guidance torque to the total steering torque. The fixed gain is set as 25% of the total steering torque in the experiment (HG-Fixed, K = 0.25).

In order to replicate the distracted state of the driver in the experiments, an additional task called rhythmic auditory serial addition was introduced. The researchers displayed a number to each driver every 30 s, and then the drivers were asked to add the current number and the previous number.

During the experiment, participants were required to complete a predefined double lane change (DLC) task. A straight segment is simulated to provide consistent driving conditions in the experiment. Participants were instructed to drive along a 300-m straight lane first, and they were instructed to maintain their position within the lane. The vehicle speed was regulated automatically by the driving simulator without driver intervention. The vehicle began to accelerate uniformly at a rate of 1.8 m/s^2^ until reaching the speed of 50 km/h, after which it maintained a constant velocity. This speed setup was designed to encourage participants to focus on their steering behaviors, particularly under the influence of haptic guidance during the lane change maneuvers.

### 2.4. Data Analysis

For the sake of determining whether there was any significant difference among the prediction models, the experimental data were statistically analyzed using one-way repeated measures analysis of variance. If the *p*-value is less than 0.05, the null hypothesis, which states that there is no significant difference, would be rejected. A *p*-value ranging from 0.05 to 0.1 would suggest a tendency towards statistical significance.

## 3. Predictive Model of Driver Steering Intention

**Figure 2:**
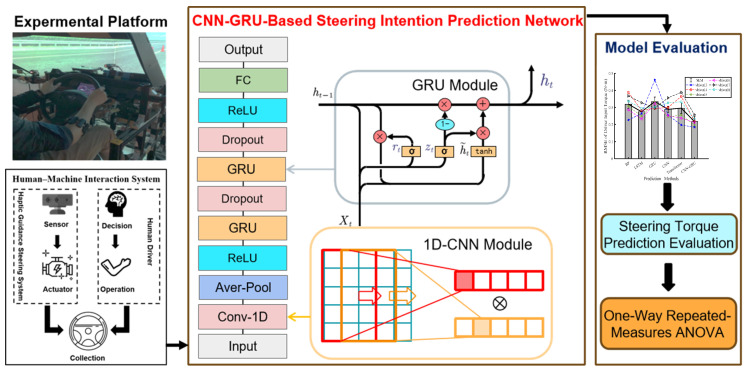
Overall structure of the driver steering intention prediction system.

This section introduces a hybrid CNN-GRU model that is designed to forecast the driver’s steering intention for human-machine shared systems of intelligent vehicles. The overall structure of the driver steering intention prediction system is shown in Figure 2.

This section firstly discusses and defines the input and output features of the steering intention prediction model. Then, the CNN module and GRU module are integrated to predict the steering intention of drivers. Finally, we discuss the selection of the evaluation measures of the hybrid CNN-GRU model to validate accuracy.

### 3.1. Feature Learning

**Figure 3:**
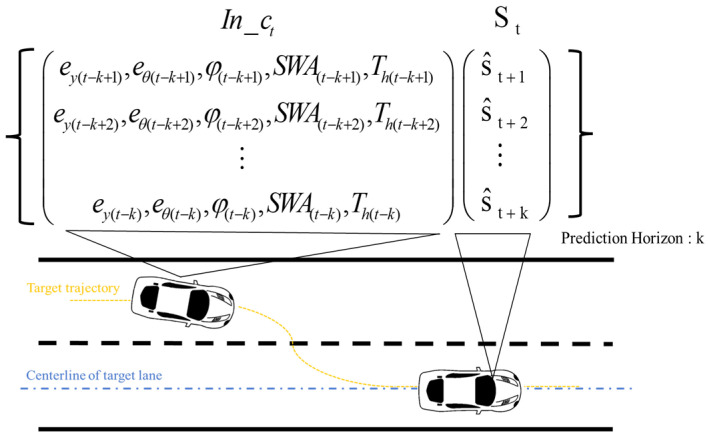
Data structure framework of input and output.

A total of 18 participants provided driving data under four conditions shown in Table 1. Information collected from six drivers formed the test data set, while the remainder served as training data. Both training and test data were randomized. Simple random sampling was applied to select data from 6 drivers to serve as the test dataset. To make multi-step predictions in the future, the data format was configured. Sequential data segmentation was performed using 10 data units, covering both the forecast and historical horizons. The structure of input and output is shown in Figure 3.

$S_{t}$ represents the anticipated sequential steering torque at time t:

(2) $$S_{t}=({\hat{s}}_{t+1},{\hat{s}}_{t+2},{\hat{s}}_{t+3}\cdots ,{\hat{s}}_{t+k})$$

where ${\hat{s}}_{t+k}$ is the predicted steering torque at time step t+k, t is the time in sequences, and *k* refers to the time step, with each step spaced 16.7 ms apart. In this paper, the value of *k* is set to 10.

Table 2 displays the input and output features of the driver steering prediction model.

Note that φ and *SWA* are used to reflect the vehicle state. $T_{h}$ represents the driver’s tactile interaction with the automated system. In addition, the output signal is the driver input torque $T_{d}$. The driver’s intention can be recognized by categorizing the steering prediction based on the variation of $T_{d}$. Therefore, the input $In\_c_{t}$ for the model can be characterized in the following manner:

(3) $$In\_c_{t}=\left(\begin{array}{l} e_{y(t-k)},e_{\theta (t-k)},\varphi_{\left(t-k\right)},SWA_{\left(t-k\right)},T_{h(t-k)},\\ e_{y(t-k+1)},e_{\theta (t-k+1)},\cdots ,SWA_{\left(t\right)},T_{h(t)} \end{array}\right)$$

The input data are normalized to stabilize the training process and eliminate the influence of outliers. Then the data are packaged in 10-step intervals according to the format in Figure 3 to enable prediction of the next 10 steps. Afterwards, the data are fed into the network.

### 3.2. The Hybrid CNN-GRU Model

A hybrid CNN-GRU model is constructed by combining CNN and GRU networks to enhance the accuracy of predicting the driver’s steering intention.

First, the CNN is responsible for extracting the characteristics of time series data. The CNN feature extraction module is composed of the following three layers: average pooling, convolutional, and ReLU. In one-dimensional data, a convolutional kernel is considered to be a feature detector and filter for removing outliers. The convolutional kernel is applied to different regions of the input data to identify the time-related interdependencies within the data. The convolutional layer produces feature maps that are sensitive to the positions of the input features. Increasing the number of feature maps elevates the dimensionality of the data, and the equation of the convolution layer is presented as:

(4) $$y_{p}^{\left(l+1\right)}=f(y_{p}^{\left(l\right)}⊗rot180(\omega_{p}^{\left(l\right)}+b_{p}^{\left(l\right)}))$$

where $y_{p}^{\left(l\right)}$ is the activation value of the l-th layer, $\omega_{p}^{\left(l\right)}$ and $b_{p}^{\left(l\right)}$ are the weight and bias matrices, respectively, and rot180(•) means to rotate the input by 180°.

To reduce the spatial size of the feature mappings, while preserving important features, a pooling layer is used to sample the feature maps after the convolutional layer. The average pooling ensures the integrity of the information. The ReLU activation function is designed to improve the learning capacity of the model in complex environments and to address the issue of vanishing gradients. The equation of the pooling layer is presented as:

(5) $$y_{p}^{\left(l+1\right)}=pooling(y_{p}^{\left(l\right)})$$

After the CNN finishes feature extraction, a GRU network adjusts the level of information updates through update and forget gates. Then the GRU propagates memory information through the unit’s hidden state $h_{t}^{j}$. At time *t*, the update of $h_{t}^{j}$ is composed of the previous hidden state $h_{t-1}^{j}$ and the candidate state ${\tilde{h}}_{t}^{j}$. Meanwhile, the update of the unit hidden state is controlled by an update gate. The procedure can be described as follows:

(6) $$h_{t}^{j}=(1-z_{t}^{j})h_{t-1}^{j}+z_{t}^{j}{\tilde{h}}_{t-1}^{j}$$

The update gate $z_{t}^{j}$ calculates the amount of information that should be preserved from the previous hidden state. If $z_{t}^{j}$ = 1, the $h_{t-1}^{j}$ ignores the previous content completely, and is computed based on the current input $x_{t}$. The calculation for the update gate is as follows:

(7) $$\;\;z_{t}^{j}=\sigma {\left(W_{z}x_{t}+U_{z}h_{t-1}\right)}^{j}$$

The candidate hidden state ${\tilde{h}}_{t}^{j}$ summarizes the information from the new input $x_{t}$ and hidden state $h_{t-1}^{j}$:

(8) $${\tilde{h}}_{t}^{j}=\tanh{\left(W_{z}x_{t}+U(r_{t}⊙h_{t+1})\right)}^{j}$$

The reset gate plays a crucial role in determining the amount of information that is forgotten about the previous hidden state. If $r_{t}^{j}$ = 1, the previous hidden state $h_{t-1}^{j}$ is updated and stored in the new memory. The reset gate can be obtained through the formula as follows:

(9) $$\;\;r_{t}^{j}=\sigma {\left(W_{r}x_{t}+U_{r}h_{t-1}\right)}^{j}$$

where $W_{\left[r,z\right]}$, $U_{\left[r,z\right]}$ are learnable parameters, σ is the Sigmoid activation function.

We employ the GRU network to enhance the precision of prediction outcomes. This section starts as a prediction module with two layers of GRUs and intersperses two dropout layers to avoid overfitting. The purpose of having the last layer as a fully connected layer is to combine the characteristic features of the steering process extracted by the preceding layers. MATLAB (R2024b) was employed for the execution of the suggested model. The output channels of the convolutional layer are configured to 10 in order to improve feature extraction. Both hidden layers of the GRU are set to 180 channels, whereas the FC layer has 10 channels. The regularization parameter is set to 0.001, while the activation functions for the GRU and convolutional layers are ReLUs. Cross-entropy error served as the output of the loss function.

### 3.3. Model Evaluation

The evaluation of the prediction performance of driver steering intention involves the use of various metrics, including the root mean square error (RMSE), mean absolute error (MAE), and mean absolute percentage error (MAPE) [39]. The *MAE* can be described as:

(10) $$\mathrm{MAE}=\frac{1}{n}\sum_{i=1}^{n}\left|{\hat{m}}_{i}-m_{i}\right|$$

The MAPE can be written as:

(11) $$\mathrm{MAPE}=\frac{1}{n}\sum_{i=1}^{n}\frac{\left|{\hat{m}}_{i}-m_{i}\right|}{{\hat{m}}_{i}}\times 100\%$$

The RMSE can be obtained as:

(12) $$\mathrm{RMSE}=\sqrt{\frac{1}{n}\sum_{i=1}^{n}{\left({\hat{m}}_{i}-m_{i}\right)}^{2}}$$

where *n* is the total number of testing sequences, i is the i-th data point, ${\hat{m}}_{i}$ and $m_{i}$ indicates the predicted value and true value, respectively.

The prediction outcomes are subjected to statistical analysis utilizing a one-way repeated measures analysis of variance with the Post Hoc test, so as to ascertain the presence of any significant disparities across the driving models. A significance threshold of *p* < 0.05 is established to reject the null hypothesis, which posits that there is no statistically significant difference. The presence of a *p*-value ranging from 0.05 to 0.1 suggests a tendency towards a noteworthy distinction.

## 4. Experimental Results

To validate the effectiveness of the suggested CNN-GRU model, we used the model to predict driver intention using experimental data involving human-machine collaboration. For comparison, a propagation (BP) network, a CNN network, an LSTM network, a GRU network, and a Transformer were also evaluated. Four experimental conditions in Table 1 were applied to obtain predictions. The five modeling methods and their parameters are defined as follows:

- LSTM Network: The LSTM network was structured with a 5-180-1 three-layer configuration, and the Adam optimization solver was employed with a learning rate of 0.005.
- BP Network: The BP network consisted of 5-6-1 three layers, and the target training accuracy was set to 0.001, which ensures model convergence and the minimum prediction error.
- CNN Network: The CNN network was made up of a 3-layer architecture, in which the convolution layers are a 1 × 10 filter size, and the fully connected layers were configured with 128 neurons for feature extraction. The learning rate of the Adam optimizer was set to 0.005 to achieve training.
- GRU Network: The GRU network was structured with three layers, arranged in a 5-180-1 configuration. The Adam optimizer was applied with a learning rate of 0.005.
- Transformer network: The Transformer network was structured with three layers, an Embedding layer, an Encoder layer, and a Decoder layer. The Adam optimizer was applied with a learning rate of 0.001.

### 4.1. Steering Torque Prediction of Attentive Drivers

**Figure 4:**
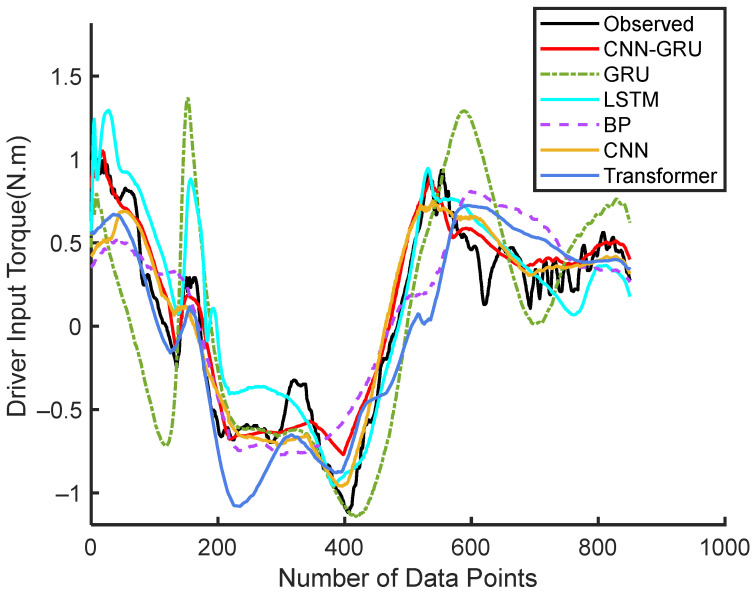
Continuous steering torque prediction results by Driver 18.

The predicted input torque for the 18th driver under experimental Condition 1 is shown in Figure 4. It is evident that the CNN-GRU model outperforms other models in terms of tracking the observed driver input torque. Its predictions exhibit the closest alignment with the actual data, showcasing higher accuracy and better tracking capabilities. Other models, such as GRU, LSTM, CNN, Transformer, and BP, deviate more significantly from the observed values, particularly in regions of rapid fluctuations and transitions.

**Figure 5:**
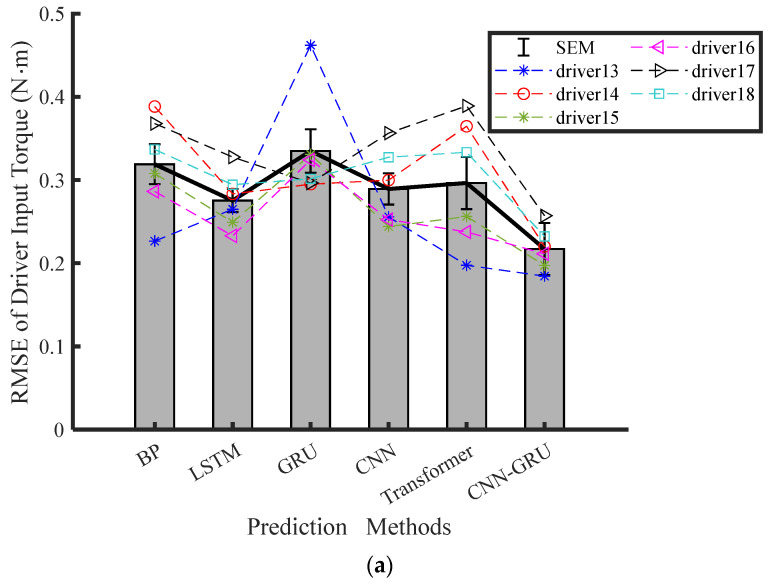

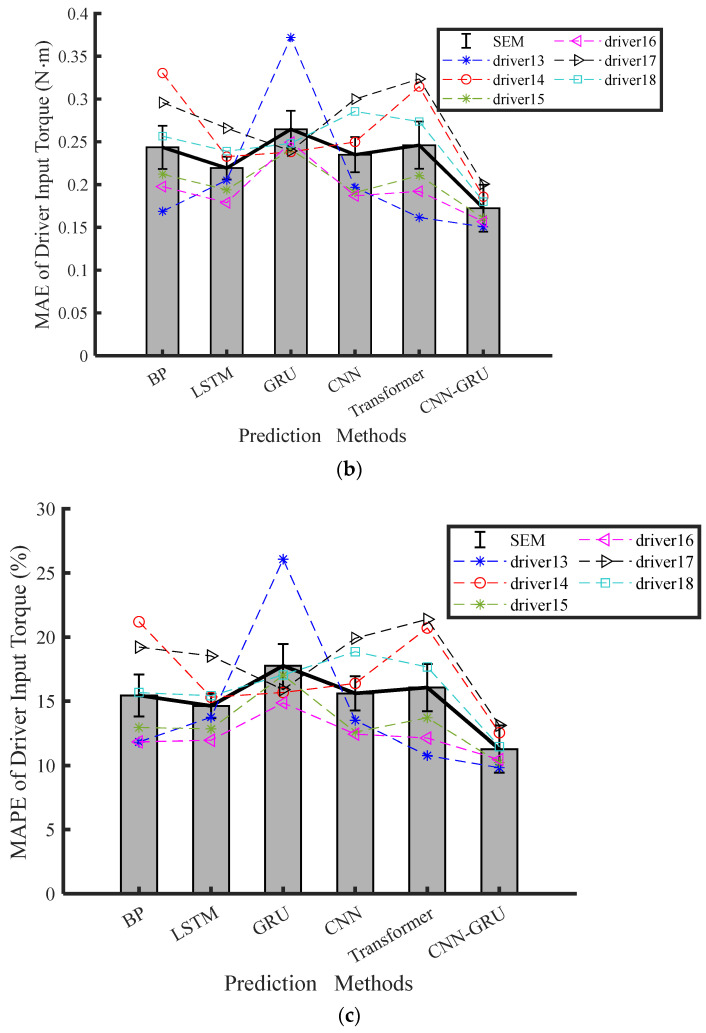
Torque prediction results for attentive driver under HG-Fixed driving conditions across four modeling methods: (**a**) RMSE, (**b**) MAE, (**c**) MPAE.

The experimental results of the final six drivers are shown in Table 3 to showcase the predictive accuracy of the proposed CNN-GRU model. The average values of RMSE, MAE, and MAPE for the steering torque prediction of the last six drivers are presented in Figure 5. According to Table 3, the CNN-GRU model achieved the least RMSE, MAE, and MAPE under Conditions 1 and 2. Based on these findings, it is clear that the CNN-GRU model is the most effective for predicting attentive driving states, as opposed to the other methods. Traditional models like GRU and LSTM primarily focus on capturing temporal dependencies but lack the ability to extract and leverage spatial features from raw input data effectively, which limits their performance for predicting driver steering intention. Due to vanishing gradients and slow convergence, it is harder for the BP network to capture both spatial and temporal features together. In addition, the GRU, LSTM, and BP methods show no significant variance from each other relative to the same performance metrics. As opposed to the BP, LSTM, GRU, and CNN networks, the CNN-GRU network significantly reduces the average RMSE of the driver input torque by 31.93%, 21.13%, 31.78%, 24.95%, and 30.65%, respectively. The CNN-GRU network reduces the average RMSE, MAE, and MAPE by 31.78%, 29.52%, and 30.02%, respectively, compared to the GRU network in Condition 2. The pairwise comparison results among different models indicate that the CNN-GRU model significantly surpasses its three counterparts in terms of RMSE, MAE, and MAPE.

### 4.2. Steering Torque Prediction of Distracted Drivers

**Figure 6:**
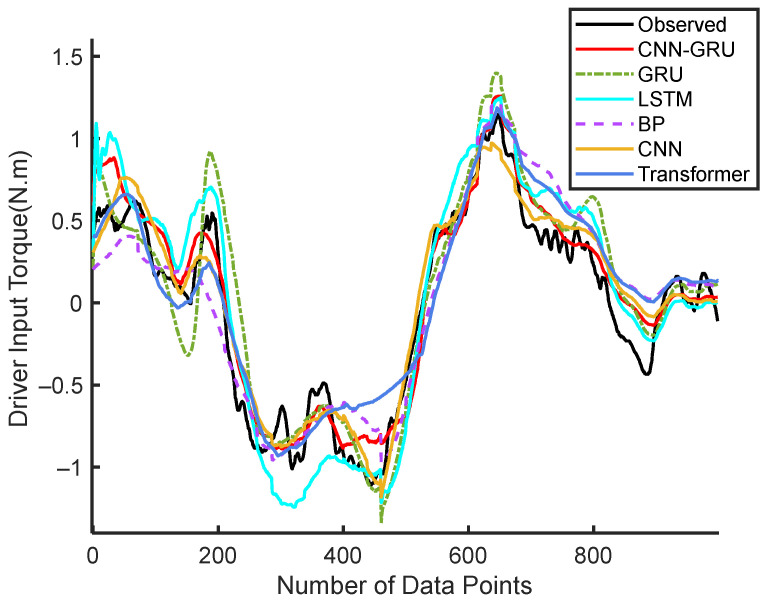
Continuous steering torque prediction results by Driver 14.

The predicted input torque for the 14th driver in a distracted state was shown in Figure 6. The CNN-GRU model demonstrated superior accuracy in predicting driver input torque compared to the other models. This higher prediction accuracy reflects the advantage of the CNN-GRU model.

**Figure 7:**
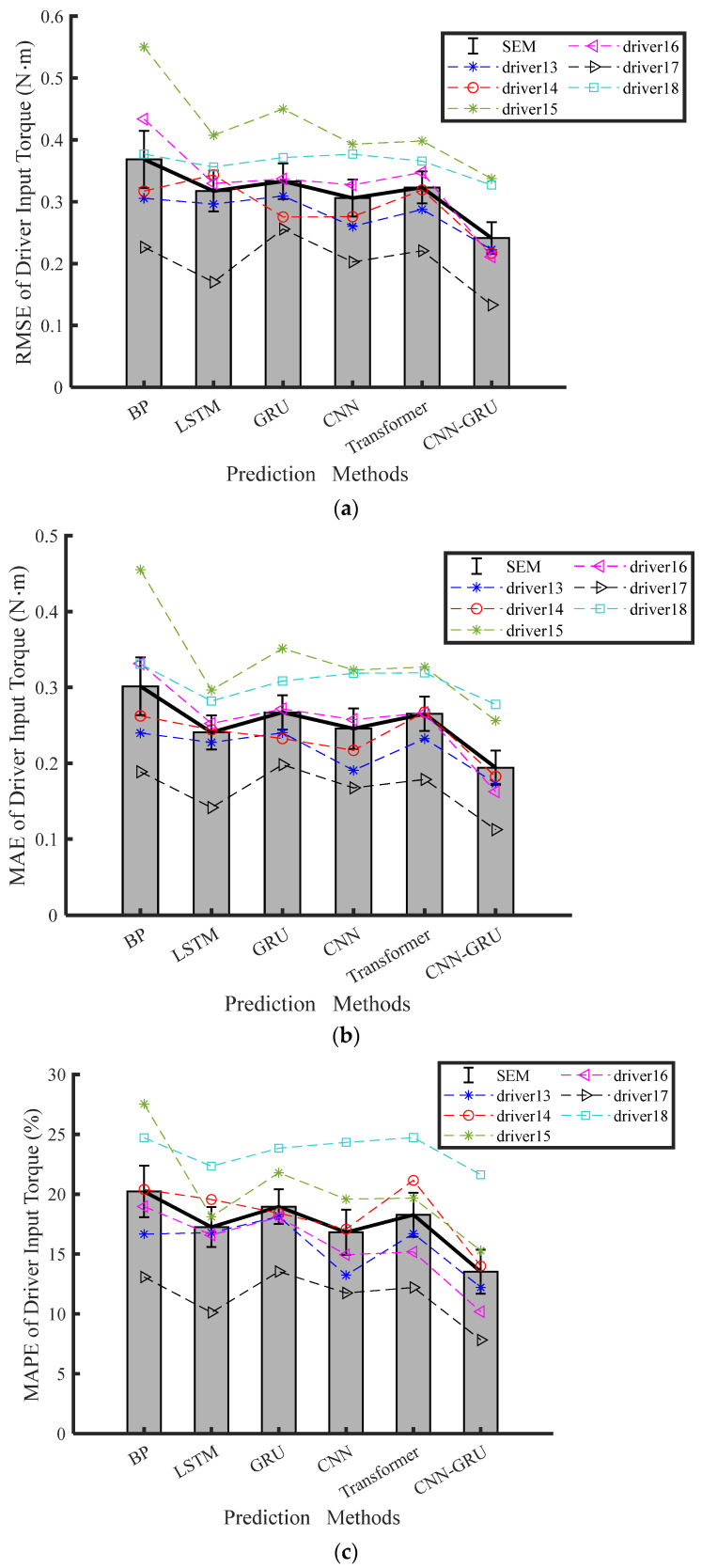
Results of torque prediction under four modeling methods during HG-adaptive driving conditions: (**a**) RMSE, (**b**) MAE, (**c**) MPAE.

Table 4 shows experimental prediction results for the steering torque prediction of the last six distracted drivers. The average values of RMSE, MAE, and MAPE are shown in Figure 7. Table 4 indicates that the prediction errors of the BP, LSTM, GRU, CNN, and Transformer models surpass the CNN-GRU model under experimental Conditions 3 and 4. Therefore, the proposed CNN-GRU model emerges as the most precise forecaster of driver input torque. On the other hand, the other models have no significant difference in terms of RMSE, MAE, and MAPE.

The CNN-GRU network, on the other hand, achieves a significant reduction in the average RMSE of the driver input torque compared to the BP, LSTM, GRU, CNN, and Transformer networks. Specifically, it decreases the average RMSE by 28.94%, 35.21%, 33.22%, 21.72%, and 27.65%, respectively. Compared with the GRU network under Condition 3, the CNN-GRU network decreases RMSE, MAE, and MAPE of the driver input torque by 33.22%, 32.33%, and 35.86%, respectively. The pairwise comparison results demonstrate that the CNN-GRU model outperforms the others by a significant margin.

The proposed CNN-GRU model was implemented on the MATLAB platform and tested on a system with an Intel Core i5—10,200H CPU (2.4 GHz) and 16 GB RAM. On average, the prediction time per sample was within 4.8–6.1 ms, which supports real-time computing in a human-machine shared control system. In comparison, baseline models such as GRU LSTM, CNN, and Transformer require approximately 8.3 ms, 10.5 ms, 11.4 ms, and 10.7 ms per sample, respectively. Specifically, our model improves the computing speed by 34.30%, 48.09%, 52.02%, and 49.06%, respectively. These results demonstrate that the proposed CNN-GRU model achieves the fastest solving speed. The automated system can conduct model training offline and directly apply model predictions to future time steps in practical applications.

In addition to superior predictive accuracy, the proposed CNN-GRU network effectively predicts intention across different driver states and haptic interactions. The model thus demonstrates strong adaptation and generalization. By combining the CNN’s capability to extract local spatial features and the GRU’s proficiency in modeling temporal dependencies, the CNN-GRU network represents the intricate inherent dynamics more comprehensively in human-machine interaction. As a result, the CNN-GRU network achieves more accurate and robust prediction of driver intentions.

To evaluate the sensitivity of the proposed model, a detailed analysis of the input window size and the number of GRU hidden units affecting prediction accuracy was conducted. The analysis data originated from a 1 h driver under distracted conditions and the HG-Adaptive scenario, and the sensitivity analysis results are shown in Table 5. It indicates that the shortest input window achieved the best performance with an RMSE of 0.1802 N·m and an MAE of 0.0997 N·m. It is obvious that the prediction error also increased with the increase in the window size. And the poorest performance is accompanied by the 20-frame window, which may be caused by the noise and redundant information of compromised feature extraction. Regarding the number of GRU hidden units, the configuration with 180 units offered an optimal balance between prediction accuracy and generalization ability. In contrast, the configuration with 60 units resulted in underfitting due to limited representation capacity, while overfitting is shown with 240 units. These findings demonstrate that the proposed CNN-GRU model is sensitive to both the temporal length of input data and the network capacity. Hence, the optimal tuning of these parameters is essential to ensure accurate prediction of driver steering intentions.

## 5. Conclusions

This article proposes a novel CNN-GRU model for predicting driver steering intention in a human-machine shared system. The proposed model extracts data features using a CNN layer and predicts the driver’s steering intention using the GRU layer. To confirm the efficacy of the suggested approach, driving simulation results are presented under four different experimental conditions. The BP, LSTM, GRU, CNN, and Transformer networks are used as performance benchmarks.

Based on the experimental results, it is evident that the proposed CNN-GRU network outperformed the other models in terms of accuracy. For example, compared with the GRU network in experimental Condition 4, the CNN-GRU network reduced RMSE, MAE, and MAPE by 27.56%, 27.34%, and 28.73%, respectively. A further benefit was the ability of the CNN-GRU network to be readily applied to differing experimental conditions, thus demonstrating strong adaptation and generalization capabilities.

However, the experiments only considered attentive and distracted states of drivers. Therefore, future research will include more intricate driver states in order to enhance the validation of the suggested prediction model’s generalizability. An additional constraint pertains to the exclusive use of a simulator platform for the execution of these investigations, and thus, we plan to conduct actual vehicle experiments to more accurately assess the effectiveness of our method.

## Figures and Tables

**Table 1:** Experimental conditions.

Condition	Driver State	Haptic Guidance
1	Attentive	HG-Fixed
2	Attentive	HG-Adaptive
3	Distracted	HG-Fixed
4	Distracted	HG-Adaptive

**Table 2:** Input and output features of the driver steering prediction model.

Data	Variable	Description
Input	$e_{y(near)}$	Lateral error at the near point (m)
	$e_{\theta (far)}$	Yaw error at the far point (deg)
	φ	Angle between the vehicle’s longitudinal axis and the current lane (deg)
	*SWA*	Steering wheel angle (deg)
	$T_{h}$	Haptic guidance torque (N⋅m)
Output	$T_{d}$	Driver input torque (N⋅m)

**Table 3:** Experimental prediction results for the steering torque prediction of the last six attentive drivers.

Variable		CNN-GRU (Ours) (1) M(SD)	GRU (2) M(SD)	LSTM (3) M(SD)	BP (4) M(SD)	CNN (5) M(SD)	Transformer (6) M(SD)	p	1–2	1–3	1–4	1–5	1–6	2–3	2–4	3–4
Condition 1	RMSE (N·m)	0.2172 (0.0257)	0.3184 (0.0261)	0.2754 (0.034)	0.3191 (0.0587)	0.2894 (0.0461)	0.3132 (0.0258)	0.000	**	**	**	**	**	0.651	1.000	0.421
	MAE (N·m)	0.1724 (0.0194)	0.2446 (0.0059)	0.2192 (0.032)	0.2436 (0.062)	0.2366 (0.0512)	0.2460 (0.0674)	0.000	**	**	*	*	*	0.845	1.000	1.000
	MAPE (%)	11.26 (0.544)	16.09 (0.341)	14.63 (0.96)	15.44 (1.631)	15.61 (3.265)	13.14 (7.747)	0.000	**	**	*	*	*	1.000	1.000	1.000
Condition 2	RMSE (N·m)	0.2287 (0.0174)	0.3119 (0.0212)	0.3552 (0.023)	0.3810 (0.020)	0.2942 (0.032)	0.3278 (0.0437)	0.000	**	**	**	*	*	1.000	*	1.000
	MAE (N·m)	0.1860 (0.0142)	0.2559 (0.0177)	0.2785 (0.017)	0.3003 (0.016)	0.2291 (0.039)	0.2665 (0.045)	0.000	***	**	**	0.293	*	1.000	**	1.000
	MAPE (%)	12.83 (1.173)	17.47 (1.303)	18.38 (1.428)	19.62 (1.324)	15.21 (3.563)	17.67 (3.742)	0.000	**	0.1897	**	0.061	*	1.000	0.197	1.000

* *p* < 0.05, ** *p* < 0.01, and *** *p* < 0.001; M denotes mean value.

**Table 4:** Experimental prediction results for the steering torque prediction of the last six distracted drivers.

Variable		CNN-GRU (Ours) (1) M(SD)	GRU (2) M(SD)	LSTM (3) M(SD)	BP (4) M(SD)	CNN (5) M(SD)	Transformer (6) M(SD)	*p*	1–2	1–3	1–4	1–5	1–6	2–3	2–4	3–4
Condition 3	RMSE (N·m)	0.2054 (0.0158)	0.3076 (0.0095)	0.3170 (0.0338)	0.2892 (0.0139)	0.2624 (0.0387)	0.2839 (0.0354)	0.000	*	*	*	*	**	1.000	1.000	1.000
	MAE (N·m)	0.1595 (0.0139)	0.2357 (0.0122)	0.2294 (0.0163)	0.2242 (0.0101)	0.2037 (0.0296)	0.2286 (0.0338)	0.000	*	**	*	0.113	0.16	1.000	1.000	1.000
	MAPE (%)	10.82 (2.44)	16.87 (1.75)	16.17 (3.38)	14.66 (1.93)	13.70 (2.41)	15.12 (2.15)	0.000	*	*	**	*	0.095	1.000	0.526	1.000
Condition 4	RMSE (N·m)	0.2413 (0.0316)	0.3331 (0.0288)	0.3171 (0.0329)	0.3684 (0.0461)	0.3159 (0.0731)	0.3231 (0.0629)	0.000	**	*	*	*	*	1.000	1.000	0.6147
	MAE (N·m)	0.1941 (0.0251)	0.2670 (0.0227)	0.2423 (0.0230)	0.3013 (0.0380)	0.2457 (0.0654)	0.2651 (0.0553)	0.000	*	*	*	0.057	**	0.4218	0.6023	0.2667
	MAPE (%)	13.52 (1.95)	18.97 (1.45)	17.26 (1.67)	20.23 (2.15)	16.82 (4.61)	18.27 (4.49)	0.000	**	*	*	*	**	0.3647	1.000	0.4931

* *p* < 0.05, ** *p* < 0.01.

**Table 5:** Sensitivity analysis results of the CNN-GRU model for the 18th driver.

Variable		RMSE (N·m)	MAE (N·m)
Windows size of input data	5	0.1802	0.0997
	10	0.2172	0.1205
	20	0.3327	0.2017
Number of GRU hidden units	60	0.2872	0.1665
	180	0.2172	0.1205
	240	0.3534	0.2413

## Data Availability

The data presented in this study are available from the corresponding author upon request.
